# Supplementary material for: Altered firing output of VIP interneurons and early dysfunctions in CA1 hippocampal circuits in the 3xTg mouse model of Alzheimer’s disease
Source: eLife. 2024 Sep 12;13:RP95412. doi: 10.7554/eLife.95412 (PMC11392531; doi:10.7554/eLife.95412)
Supplement: Supplementary file 1. — Notes: (1) The reported n corresponds to the number of animals, cells (underlined), or slices (italic). (2) Animals of both sexes with equal distribution were used throughout the study. [file elife-95412-supp1.docx]

**Supplementary File 1. Summary table of statistical analysis conducted throughout the study.**

| **Figure** | **Condition** | **n** | **Test** | ***P* value** |
| --- | --- | --- | --- | --- |
| Fig 1a | 6E10+ cell density: 3-month vs. 6-month | 3,*8* vs*.* 3,*8* | Wilcoxon Rank | 0.1027 |
| Fig 1d | VIP+ cell density: VIP-nonTg vs. VIP-Tg  CR+ cell density: VIP-nonTg vs. VIP-Tg  VIP+/CR+ cell density: VIP-nonTg vs. VIP-Tg | 3,*17* vs. 3,*13*  3,*11* vs. 3,*11*  3,*11* vs. 3,*11* | Wilcoxon Rank  Wilcoxon Rank  Wilcoxon Rank | 0.4442  0.8720  0.0751 |
| Fig 1 - S1b | RI Sample: VIP-nonTg vs. VIP-Tg  RI Test: VIP-nonTg vs. VIP-Tg | 10 vs. 11  10 vs. 11 | Unpaired t-test  Unpaired t-test | 0.8373  0.0040** |
| Fig 1 - S1c | Total object exploration time (Test): VIP-nonTg vs. VIP-Tg  Total horizontal activity (Test): VIP-nonTg vs. VIP-Tg | 10 vs. 11  10 vs. 11 | Unpaired t-test  Unpaired t-test | 0.1408  0.0933 |
| Fig 2c | AP amplitude: VIP-nonTg vs. VIP-Tg  AP half-width: VIP-nonTg vs. VIP-Tg  AP depolarization rate: VIP-nonTg vs. VIP-Tg  AP repolarization rate: VIP-nonTg vs. VIP-Tg  AP area: VIP-nonTg vs. VIP-Tg | 5,8 vs. 3,10  5,8 vs. 3,10  5,8 vs. 3,10  5,8 vs. 3,10  5,8 vs. 3,10 | LMM  LMM  LMM  LMM  LMM | 0.843  0.018*  0.461  0.024*  0.025* |
| Fig 2d | AP amplitude: VIP-nonTg vs. VIP-Tg  AP half-width: VIP-nonTg vs. VIP-Tg  AP area: VIP-nonTg vs. VIP-Tg | 5,7 vs. 3,7  5,7 vs. 3,7  5,7 vs. 3,7 | Two-way ANOVA  Two-way ANOVA  Two-way ANOVA | <0.0001****  <0.0001****  <0.0001**** |
| Fig 2e | Norm. AP depol. rate: VIP-nonTg vs. VIP-Tg  Norm. AP repol. rate: VIP-nonTg vs. VIP-Tg | 5,7 vs. 3,7  5,7 vs. 3,7 | Two-way ANOVA  Two-way ANOVA | 0.9998  <0.0001**** |
| Fig 2f | AP number: VIP-nonTg vs. VIP-Tg | 5,7 vs. 3,7 | Two-way ANOVA | 0.0264* |
| Fig 2 - S1c | sIPSC amplitude: VIP-nonTg vs. VIP-Tg  sIPSC frequency: VIP-nonTg vs. VIP-Tg  sEPSC amplitude: VIP-nonTg vs. VIP-Tg  sEPSC frequency: VIP-nonTg vs. VIP-Tg | 5, 7 vs. 5, 6  5, 7 vs. 5, 6  5, 5 vs. 4, 4  5, 5 vs. 4, 4 | Unpaired t-test  Unpaired t-test  Unpaired t-test  Unpaired t-test | 0.4664  0.3349  0.2961  0.5242 |
| Fig 2 - S2b | IS3 soma area: VIP-nonTg vs. VIP-Tg | 5, 6 vs. 4, 5 | Wilcoxon Rank | 0.4818 |
| Fig 2 - S2c | IS3 dend. surface: VIP-nonTg vs. VIP-Tg | 5, 6 vs. 4, 5 | Wilcoxon Rank | 0.3773 |
| Fig 2 - S2d | IS3 dend. length: VIP-nonTg vs. VIP-Tg | 5, 6 vs. 4, 5 | Wilcoxon Rank | 0.3773 |
| Fig 2 - S2e | IS3 dend. branch points: VIP-nonTg vs. VIP-Tg | 5, 6 vs. 4, 5 | Wilcoxon Rank | 0.7258 |
| Fig 2 - S2f | IS3 dend. length vs. distance from soma:  VIP-nonTg vs. VIP-Tg  IS3 dend.number of intersections vs. distance from soma: VIP-nonTg vs. VIP-Tg  IS3 dend. number of nodes vs. distance from soma: VIP-nonTg vs. VIP-Tg | 5, 5 vs. 4, 5  5, 5 vs. 4, 5  5, 5 vs. 4, 5 | Two-way ANOVA  Two-way ANOVA  Two-way ANOVA | >0.9999  0.9941  >0.9999 |
| Fig 3b | sIPSC amplitude: nonTg vs. Tg  sIPSC frequency: nonTg vs. Tg | 5, 16 vs. 4, 10  5, 16 vs. 4, 10 | Wilcoxon Rank  Wilcoxon Rank | 1.0000  0.0017** |
| Fig 3c | sIPSC rise time: nonTg vs. Tg  sIPSC decay tau: nonTg vs. Tg  sIPSC charge transfer: nonTg vs. Tg | 5, 16 vs. 4, 10  5, 16 vs. 4, 10  5, 16 vs. 4, 10 | Wilcoxon Rank  Wilcoxon Rank  Wilcoxon Rank | 0.0095**  0.0017**  0.0214* |
| Fig 3e | VGAT bouton density: nonTg vs. Tg  VGAT/CR bouton density: nonTg vs. Tg | 4,*19* vs*.* 4,*21*  4,*19* vs*.* 4,*21* | Unpaired t-test  Wilcoxon Rank | 0.8886  0.9039 |
| Fig 3 - S1b | sEPSC amplitude: nonTg vs. Tg  sEPSC frequency: nonTg vs. Tg | 5, 8 vs. 4, 5  5, 8 vs. 4, 5 | Wilcoxon Rank  Wilcoxon Rank | 0.3185  0.4789 |
| Fig 3 - S1c | sEPSC rise time: nonTg vs. Tg  sEPSC decay tau: nonTg vs. Tg  sEPSC charge transfer: nonTg vs. Tg | 5, 8 vs. 4, 5  5, 8 vs. 4, 5  5, 8 vs. 4, 5 | Wilcoxon Rank  Wilcoxon Rank  Unpaired t-test | 0.6722  0.7782  0.4585 |
| Fig 4c | Animal speed: nonTg vs. Tg  Animal mobility rate: nonTg vs. Tg  Total distance: nonTg vs. Tg  Rearing: nonTg vs. Tg | 13/group  13/group  13/group  9 vs. 7 | Wilcoxon Rank  Wilcoxon Rank  Wilcoxon Rank  Wilcoxon Rank | 0.9398  0.4637  0.7432  0.4990 |
| Fig 4e | Walk AVG Z-score: nonTg vs. Tg  Walk Peak Z-score: nonTg vs. Tg | 9 vs. 8  9 vs. 8 | Wilcoxon Rank  Wilcoxon Rank | 0.9254  0.6732 |
| Fig 4g | Alternation-%: nonTg vs. Tg | 9 vs. 6 | Fisher’s Exact | 0.2517 |
| Fig 4h | nonTg AVG Z-score stem vs. D-zone  Tg AVG Z-score stem vs. D-zone  nonTg Peak Z-score stem vs. D-zone  Tg Peak Z-score stem vs. D-zone | 5  5  5  5 | Wilcoxon Signed  Wilcoxon Signed  Wilcoxon Signed  Wilcoxon Signed | 0.3125  0.0313*  0.625  0.0313* |
| Fig 4i | Object entries: nonTg vs. Tg  Object time: nonTg vs. Tg | 10 vs. 8  10 vs. 8 | Wilcoxon Rank  Wilcoxon Rank | 0.7700  0.6676 |
| Fig 4j | Object Sample AVG Z-score: nonTg vs. Tg | 10 vs. 9 | Wilcoxon Rank | 0.0152* |
| Fig 4 - S1b | Home Cage CaT peak amplitude: nonTg vs. Tg  Home Cage CaT frequency: nonTg vs. Tg | 9 vs. 8  9 vs. 8 | Wilcoxon Rank  Wilcoxon Rank | 0.3539  0.0915 |
| Fig 4 - S1d | Locomotion Z-score: nonTg vs. Tg  Immobility Z-score: nonTg vs. Tg  Rearing Z-score: nonTg vs. Tg | 9 vs. 8  9 vs. 8  9 vs. 8 | Wilcoxon Rank  Wilcoxon Rank  Wilcoxon Rank | 0.4257  0.8721  0.2259 |
| Fig 4 - S1e | DZ average speed: nonTg vs. Tg  DZ average mobility speed: nonTg vs. Tg  DZ distance traveled: nonTg vs. Tg | 10 vs 5  10 vs 5  10 vs 5 | Wilcoxon Rank  Wilcoxon Rank  Wilcoxon Rank | 0.7229  0.1462  0.2298 |
| Fig 4 - S1f | Stem average speed: nonTg vs. Tg  Stem average mobility speed: nonTg vs. Tg  Stem distance traveled: nonTg vs. Tg | 10 vs 5  10 vs 5  10 vs 5 | Wilcoxon Rank  Wilcoxon Rank  Wilcoxon Rank | 0.6359  0.2298  0.6359 |
| Fig 5b | Home Cage CaT peak amplitude: nonTg vs. Tg  Home Cage CaT frequency: nonTg vs. Tg | 4 vs. 5  4 vs. 5 | Wilcoxon Rank  Wilcoxon Rank | 0.8166  0.8166 |
| Fig 5d | Walk Peak Z-score: nonTg vs. Tg  Walk AUC Z-score: nonTg vs. Tg | 4 vs. 5  4 vs. 5 | Wilcoxon Rank  Wilcoxon Rank | 0.4832  0.3464 |
| Fig 5f | nonTg AVG Z-score stem vs. D-zone  Tg AVG Z-score stem vs. D-zone  nonTg Peak Z-score stem vs. D-zone  Tg Peak Z-score stem vs. D-zone | 4  5  4  5 | Paired t-test  Wilcoxon Signed  Paired t-test  Wilcoxon Signed | 0.0335*  1  0.0333*  1 |
| Fig 5h | Object-Zone AVG Z-score: nonTg vs. Tg  Object-Zone RMS Z-score: nonTg vs. Tg | 4 vs. 5  4 vs. 5 | Wilcoxon Rank  Wilcoxon Rank | 0.7584  0.0107* |
